# The 2017/18 Health Behaviour in School-aged Children (HBSC) study – Methodology of the World Health Organization’s child and adolescent health study

**DOI:** 10.25646/6904

**Published:** 2020-09-16

**Authors:** Irene Moor, Kristina Winter, Ludwig Bilz, Jens Bucksch, Emily Finne, Nancy John, Petra Kolip, Lisa Paulsen, Ulrike Ravens-Sieberer, Marina Schlattmann, Gorden Sudeck, Catherina Brindley, Anne Kaman, Matthias Richter

**Affiliations:** 1 Martin Luther University Halle-Wittenberg Medical Faculty, Institute of Medical Sociology; 2 Brandenburg University of Technology Cottbus-Senftenberg Faculty of Social Work, Health Care and Music, Institute of Health; 3 Brandenburg University of Technology Cottbus-Senftenberg Faculty of Health Sciences; 4 University of Education Heidelberg Faculty of Natural and Sociological Sciences, Department of Prevention and Health Promotion; 5 Bielefeld University School of Public Health, Department Prevention and Health Promotion; 6 University Medical Center Hamburg-Eppendorf Center for Psychosocial Medicine, Department of Child and Adolescent Psychiatry, Psychotherapy and Psychosomatics; 7 Eberhard Karls University of Tübingen Institute of Sport Science; 8 Eberhard Karls University of Tübingen Interfaculty Research Institute for Sport and Physical Activity

**Keywords:** ADOLESCENTS, HEALTH BEHAVIOUR, HEALTH MONITORING, HEALTH DETERMINANTS, HBSC

## Abstract

The Health Behaviour in School-aged Children (HBSC) study is an international research project in collaboration with the World Health Organization (WHO) for over 35 years. HBSC is the largest study on child and adolescent health and one of the most important sources of data for the WHO’s international comparative health monitoring. Every four years, data on the health and health behaviour of students aged 11, 13 and 15, as well as the social contexts and conditions for growing up healthy, are collected. A total of 50 countries belong to the HBSC network, with 45 countries taking part in the 2017/18 survey. Germany has contributed to the HBSC surveys since 1993/94. For the most recent 2017/18 cycle, students at 146 schools in Germany were interviewed (response rate of schools: 15.6%). A net sample of n = 4,347 girls and boys was achieved for Germany (response rate: 52.7%). Participation was voluntary and the survey was conducted in German school years five, seven and nine (corresponding to ages 11, 13 and 15). A weighting procedure was applied to allow for representative findings on the health of children and adolescents in Germany. HBSC offers a valuable contribution to health monitoring and provides numerous starting points to identify needs, risk groups and fields of action to initiate targeted and actual needs-based measures of prevention and health promotion in the school setting.

## 1. Background

### HBSC – The international World Health Organization study

For adult-age health, childhood and adolescence are determining life phases. A string of publications in the journal ‘The Lancet’ [1–4], as well as reports by the World Health Organization (WHO) [5–9] and the United Nations Children’s Fund (UNICEF) [10–13] all highlight the relevance of child and adolescent health for public health policies and practice, and science’s mandate to provide an encompassing data basis to support decision makers in developing health promotion and prevention measures.

Considering the number of participating countries, the Health Behaviour in School-aged Children study – abbreviated to HBSC – is one of the world’s largest studies on child and adolescent health, and an important basis of data for international comparative health reporting in childhood and adolescence by the WHO [9, 14]. The study aims to provide data on the health and health behaviour of students aged 11, 13 and 15 years, as well as the general conditions required to grow up healthy.

The HBSC study is an international collaborative research project that has received WHO support for over 35 years. Scientists from the United Kingdom, Finland and Norway started the study in 1982. The first survey cycle was conducted in 1983/84 in the founding member countries as well as in Austria and Denmark. Since 1985/86, there is a survey every four years in a successively increasing number of countries. 50 countries now make up the HBSC network. The 2017/18 survey was conducted in 45 countries and involved the participation of over 200,000 adolescents [9].

**Figure 1:**
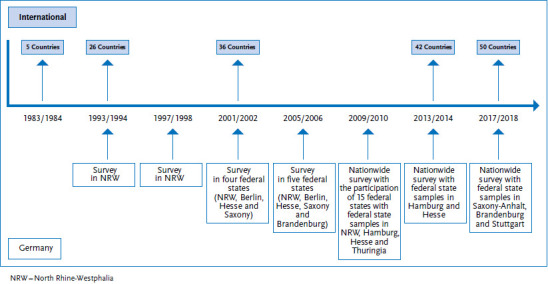
Number of countries in the HBSC network and participation of Germany by survey cycle Source: Own diagram

In terms of Germany’s involvement, the most populous federal state of North Rhine-Westphalia first took part in the 1993/94 cycle of the HBSC study. In the 1997/98, 2001/02 and 2005/06 cycles, Saxony, Hesse, Berlin, Thuringia and Hamburg also took part. With the exception of Baden-Württemberg, all federal states then took part in the 2009/10 cycle. Since the 2013/14 cycle, all 16 federal states have collected data. The current 2017/18 cycle was conducted with two supplementary samples in Brandenburg and Saxony-Anhalt, as well as a complete urban survey for the city of Stuttgart (Figure 1).

**Info box:**

**German secondary school system**

This paper includes terminology specific to the German secondary school system, whereby students can attend different schools that vary in their level of academic and/or vocational focus. In general, a Hauptschule is attended by students aged 10 to 16 and offers a basic general education, a Realschule provides a more extensive education for students aged between 10 and 16. A Gymnasium teaches students aged between 10 and 19, provides an in-depth general education and is focused on preparing students for higher education.

Gemeinschaftschulen are secondary education schools, primarily for students aged 10 to 16, where students learn together and are able to sit the same qualifications offered in the three other school types (Hauptschule, Realschule and Gymnasium).

The 2017/18 HBSC cycle in Germany was accomplished by the following universities: Martin Luther University of Halle-Wittenberg (managed and co-ordinated by Prof. Dr Matthias Richter); Brandenburg University of Technology Cottbus-Senftenberg (Prof. Dr Ludwig Bilz); Heidelberg University of Education (Prof. Dr Jens Bucksch); University of Bielefeld (Prof. Dr Petra Kolip); Eberhard Karls University Tübingen (Prof. Dr Gorden Sudeck); and the University Medical Center Hamburg-Eppendorf (Prof. Dr Ulrike Ravens-Sieberer).

The HBSC study has three main strengths: 1) the international comparability of data; 2) the potential to analyse trends and contributions to health monitoring; and 3) the availability of representative and broad data on child and adolescent health and health behaviour, as well as on factors from the social and school environments that potentially influence health. HBSC can therefore provide an important contribution to health monitoring as well as to national and international health targets. This information offers the scope to identify risk factors and fields of action, shed light on currently unmet needs and to indicate focuses for prevention and health promotion, particularly in the school setting [15].

## 2. Methodology

### 2.1 HBSC topics and HBSC instruments

For the HBSC study, self-reported data of adolescents across Germany are collected via a questionnaire (paper-and-pencil method). The social determinants of health and health behaviour were focus of the questionnaire. HBSC is conducted using standardised, internationally co-ordinated guidelines in all participating countries [6, 9]. To ensure international comparability, the same core questionnaire is used in all countries. In addition, depending on a country’s respective interests, further optional modules exist which can be used. All questions are continuously developed and validated. For the current survey cycle, the German questionnaire was tested in a national pre-test in two school classes of 11-year-old children (school year five in Germany) at two different school types (secondary school (‘Oberschule’) and grammar school (‘Gymnasium’), Info box). The ‘Oberschule’ is a special type of school in Saxony. ‘Oberschulen’ bring together lower secondary and intermediate secondary education under one roof and are orientated towards occupational training’ [16].

Based on the pre-test, it was examined how long it took for students to complete the questionnaire and ambiguities with regard to the wording as well as issues related to how readily understandable the questions were noted. Some difficulties were solved by introducing short notes explaining the questions, yet without changing the validated items themselves. For this, the international HBSC study group regularly detects important issues for adolescents (via the Youth Engagement Advisory Group) and uses this information to further develop the study. The international protocol, which can be obtained on demand from the HBSC study group, contains detailed information on methodology, items and scales, as well as validation [6, 17]. Table 1 provides an overview of the topics covered by the survey.

### 2.2 Survey design and sample

HBSC is designed as a cross-sectional study. The target populations are children and adolescents in the age groups of 11, 13 and 15 with a mean deviation of 0.5 years. In Germany, these age groups correspond roughly to school years five, seven and nine. According to the HBSC guidelines, the target number of participants per age group is n=1,500, leading to a total net sample of N=4,500.

Germany’s national sample is a cluster sample, which means that surveying units are primarily schools and, secondly, school classes. Sampling was conducted using the IBM SPSS Statistics 25 software with a PPS design (probability proportional to size) which took into account school size and the distribution (in percentage) of students for the included grades in the corresponding federal state, stratified by type of school. The culture and/or education ministries of each federal state were asked to provide the most up-to-date list of all types of general education schools in the respective federal state. Privately run schools and schools for children with special needs were not considered. Based on these lists of schools, a sample was drawn that corresponds to the percentage distribution of students by federal state, type of school, grade and sex. Federal states with large populations and a correspondingly high number of students, such as North Rhine-Westphalia and Bavaria, are represented with a higher percentage (in line with the federal distribution) than federal states with considerably fewer students (such as Bremen or Saarland). In a second step, based on the percentage distribution of students at the different school types in each state, the required number of schools was then randomly selected. Where this information was available, sampling took into account the size of schools and the number of students in the relevant grades. Due to low response rates in some federal states, further samples were drawn where necessary.

Considering the experiences gained from previous HBSC cycles and based on other school surveys, a response rate of 20% for schools and 70% for students in Germany was anticipated. These estimates already took into account that participation rates could be lower than during the 2013/14 HBSC cycle. In order to avoid distortions and ensure the international comparability of data, data cleaning eliminated students from the data set who were either more than six months older or younger than the surveyed age groups of 11-, 13- and 15-year-olds. Next to response rates, a 20% buffer for this data cleaning was taken into account to ensure the necessary size of the sample.

### 2.3 Recruiting schools and survey implementation

In Germany, the HBSC study was approved in advance by the education ministries of each federal state (with the exception of North Rhine-Westphalia, where the decision lay with the school administration) and, depending on state regulation, in consultation with the state’s data protection officer. The German survey team in Halle planned and co-ordinated the standardised survey, while school recruitment was conducted de-centrally at all HBSC survey locations in Germany.

All randomly drawn schools received a letter from the German national HBSC team inviting them to participate in the survey. Schools that did not respond to this letter were contacted again, either through a further letter or a telephone call. Participating schools received the interview materials and an information flyer in advance to provide teachers, parents and students with a broad basis of information on the study. Details on the content of the study, data protection and contact persons were provided. In case of any questions, all study participants (schools, teachers, parents and students) could contact the national HBSC team, as well as receive information on the study website, where, with the appropriate password, they could also access the questionnaire.

At each participating school, a fifth-, seventh- and ninth-year class was invited to take part. Schools randomly selected classes for participation. In order to standardise surveying at schools, schools and teachers received detailed information and instructions for the survey day. Schools and especially teachers decided the date on which the survey would take place depending on activities already scheduled at their schools. Students who provided the consent of their legal guardians and wished to take part were handed out the questionnaire in class. In addition to the questionnaire, participants received an empty envelope with university stamp into which they placed the questionnaire after completion, sealing the envelope before collection. All data was surveyed anonymously. Filling out the questionnaire took around 45 minutes. Collected questionnaires were sent with pre-addressed packages to the national HBSC centres. Specific regulations of the ministry of education in Saxony meant that surveying there was conducted by members of the HBSC team. Data was surveyed in all federal states between February and September 2018.

### 2.4 Data protection and ethics

The survey was conducted anonymously and developed to comply with the EU’s General Data Protection Regulation (GDPR) and Germany’s Data Protection Act (BDSG). A corresponding concept was developed with the data protection officer of Martin Luther University Halle-Wittenberg. Furthermore, the study received the approval of the Ethics Committee of the General Medical Council Hamburg (processing code PV5671). Participation in the study was voluntary for schools and students. Schools, children and adolescents could refuse to participate or withdraw their consent until the day of the survey. Moreover, all participating students were free to cease filling out the questionnaire at any moment, or to answer only selected questions. Active consent to participate in the study was sought from parents or legal guardians and students which was checked by teachers. Schools participated in the study at no financial cost. An external services provider conducted data entry for all federal states centrally based on standardised provisions and quality-assured procedures, such as entering the data twice and pre-defining valid entries. Following quality assurance, the services provider shredded questionnaires.

It is impossible to identify schools or individual students using the data as no names were associated to the questionnaire data. To ensure the total pseudonymisation of the data provided, the list of participating schools was eliminated from the random sample immediately after the completed questionnaires were received. Schools shredded the active consent forms received from parents or legal guardians and students at the end of the survey in accordance with Germany’s DIN norm 6639.

## 3. Response and representativeness

### 3.1 Comparisons between the unadjusted and adjusted gross samples

Applying standardised procedures, the Data Management Center in Bergen cleaned the data received from a total of 6,097 students in Germany. Students outside of the accepted variance of ±0.5 years from the realised sample of 11, 13 and 15 years were excluded. A small number of further cases were excluded because no sex was reported. A total of n=1,750 cases (28.7%) were removed from the data set. These cases can be considered as quality neutral losses. The net sample therefore consisted of n=4,347 girls and boys.

### 3.2 Case numbers and response rates

Overall, 146 schools were included in the HBSC study, which corresponds to an average response rate among schools of 15.6%. The response rate for students was 52.7% on average. Depending on the federal state, response rates between 39.2% and 76.3% were achieved on student level. Other health surveys concerning schools have achieved similar or higher response rates for schools and students [18]. Compared to previous HBSC study cycles, the response rates of schools, and of students in particular, have decreased. The main reasons stated by schools – either in writing or by phone – were that they lacked the capacities, had too few teaching staff due to high rates of sickness, had already agreed to participate in other studies, or they pointed to the increasing number of requests to participate in studies they had received in recent years. The reasons most frequently stated for non-participation by students were sickness, lack of interest or the absence of consent from parents or legal guardians. Europe’s new general data protection regulation also caused a certain level of uncertainty and reservations, in particular among parents. A certain degree of ‘survey fatigue’ was also observed due to the increasing number of surveys among students at schools. A further problem was that for parents without sufficient proficiency in German, the information flyer was hard to understand. These problems and reasons for non-participation were also expressed in other studies and/or surveys at schools [19].

### 3.3 Composition of the realised sample

The final data set was compiled via a multi-step process. At first, partial samples from the representative samples for Brandenburg, Saxony-Anhalt and the city of Stuttgart were integrated into the national data set. This national data set was then processed by the HBSC Data Management Centre (DMC) at the University of Bergen in Norway. Following a set and standardised procedure, the national data of all participating HBSC countries was tested for incorrect entries (particularly with regard to age) and cleansed. A harmonisation of age groups enables international comparisons. Table 2 provides information on the final data set for Germany stratified by federal state, number of schools and sex.

For each federal state, sample selection was stratified by the existing school types. Due to the heterogeneous structures that exist in the 16 federal states, the types of schools were split into four categories to allow for comparisons (Table 3).

### 3.4 Weighting

The realised sample was compared with the target sample regarding a representative distribution by federal state and type of school. Here, slightly lower participation rates in some federal states or by certain types of schools, as well as data cleansing, have led to small incongruences in the composition of the realised sample. Therefore, a weighting variable was applied. The variable corrects the realised proportions with regard to the target distribution by federal state, type of school, sex and age, and thereby improves the representativeness of the sample. All analyses were conducted with the weighting variable; absolute number of cases are presented unweighted.

### 3.5 Operationalisation of sociodemographic and socioeconomic variables

Student’s age was operationalised through the data provided in the survey on year and month of birth. Plausibility checks were used to complement data from students who failed to report their age based on the data provided on school year. School year was also surveyed and students could answer either five, seven or nine. Sex was surveyed by asking whether the respondent was a girl or a boy.

To operationalise the socioeconomic status of adolescents, a number of indicators, such as type of school, and the family affluence of children and adolescents, were used. Type of school was not surveyed, this data was provided by the schools themselves when they sent back the survey questionnaires. Due to the highly heterogeneous nature of school forms across all federal states, these were categorised into either four (grammar, intermediate and lower secondary schools, as well as mixed forms), or two groups (grammar schools, other types of schools) during analysis. Only the grammar schools are comparable across all federal states; all other types of school have different specific characteristics in each federal state. It remains unclear whether the differences found by type of school are owed to the school form as such or to the differences between federal states [20].

In general, operationalising socioeconomic status during adolescence is challenging. Usually the socioeconomic status of adolescents is surveyed by using family indicators, such as parents’ levels of education, occupation or income. However, it is unsure whether these indicators – in particular with increasing age of adolescents – can properly indicate the socioeconomic position of adolescents themselves. Furthermore, children and adolescents find it difficult to report this information on their parents correctly, resulting in missing or incorrect values. Adequate forms of indicators for adolescents are increasingly being discussed [21–24]. The HBSC study has therefore developed the Family Affluence Scale (FAS), which reflects family affluence and is easy for students to complete [25–28]. Over the last 20 years, this instrument has been continuously adapted to the constantly changing living conditions of children and adolescents [28, 29]. In the 2013/14 and 2017/18 survey cycles, FAS was operationalised based on six items (car ownership, own (bed) room, holidays with family, having a computer, number of bathrooms, owning a dishwasher). Points are given for each item and added up. A relative measure was used for the analyses, which led the FAS to be divided into three categories indicating either low (lowest 20% of the sample), medium (middle 60% of the sample) and high (highest 20% of the sample) family affluence [6, 28].

HBSC also surveyed the migration background of students, whereby students were asked to state where they, their mother and their father had been born. Due to data protection regulations, an open question was not possible, which meant that adolescents were asked to choose from a short list of countries that included Germany or they could select ‘other country’. Similar to the German Health Interview and Examination Survey for Children and Adolescents (KiGGS) [30], migration background was split into three categories based on the reported countries of birth of students and their parents. Adolescents with one parent born outside of Germany are categorised as having a one-sided migration background. A two-sided migration background was present if a) the adolescent itself was not born in Germany and at least one parent was not born in Germany or b) both parents had moved to Germany and were not born in Germany.

All other adolescents were considered as having no migration background. Table 4 shows the frequencies and percentage distribution of these sociodemographic and socioeconomic variables.

## 4. Discussion

The HBSC study provides internationally comparable, valid and representative data on child and adolescent health in Germany. Standardised surveying procedures across all participating countries and subsequent data cleaning make this data an important basis of information on the health of children and adolescents in up to 50 countries. In some countries, HBSC data is the only comprehensive source of data on child and adolescent health. The HBSC study covers numerous important determinants for the health and health behaviour of adolescents and, moreover, analyses possible influencing factors against the backdrop of family, school and peer group. In collaboration with the WHO, which uses the HBSC data as an important fundament to assess child and adolescent health, the international results are presented following a survey cycle every four years [9, 31, 32].

In the past, the data was also used to conduct more in-depth analyses, such as reports on trends and differences in levels of alcohol consumption in the WHO Europe region [5], or on obesity [7]. HBSC also co-operates with the United Nations children’s fund (UNICEF). This work has led to a number of international analyses on child and adolescent health, such as on inequality of health opportunities [10–13], trends for life satisfaction and health [33] or on diet and physical activity [34] relative to family affluence.

## Limitations

In spite of the study’s importance and its international presence, participation rates have been dwindling in many countries, including Germany, both for schools and for students. A broad set of measures to attract participants has not reversed this long-term trend of decreasing participation rates, which has also been observed in other studies. Reasons for this decreasing interest of schools could be the increasing number of school surveys, a lack of staff, or that schools are not provided with incentives, for example in the form of specific school evaluations (with the exception of the representative federal state level samples from Brandenburg and Saxony-Anhalt). Due to data protection concerns such an approach is not possible in all federal states, and would also require a considerably higher number of academic staff and funding.

Models could, for example, be HBSC countries that achieve higher participation rates due to seeking only ‘passive consent’. Passive consent means that participants are considered to have provided consent if they do not actively object to participating. The opposite would be ‘active consent’, i.e. participants need to actively provide consent to participate. The German HBSC study is based on active consent.

Studies have shown that under active consent, as opposed to passive consent, not only were participation rates lower, but that also far fewer male and older participants were reached [35, 36]. The results on reported health behaviour are heterogeneous using active consent. Whereas one study, for example, showed lower prevalence for risky, as well as for anti-social or deviant behaviour [35], a meta analysis on substance consumption showed higher prevalence [36]. Moreover, participation in studies such as the Programme for International Student Assessment (PISA) is compulsory for schools in Germany and, therefore, has very high participation rates [37]. In future, it is also possible that online surveys could generate higher rates of participation while requiring fewer resources. Other studies have already shown a greater preference for online formats when both formats (paper-and-pencil method vs. online) are offered [18].

The cross-sectional design of the HBSC study presents a further limitation. Such a design is well suited for trend analyses, but not to the deduction of causalities. Furthermore, HBSC uses a broad set of indicators to survey child and adolescent health, yet this has the disadvantage that only few items could be used for specific topics. A further limitation of the questionnaire on gender identity is that it does not use a third category such as ‘other’. This should be changed in future HBSC survey cycles.

## Outlook

For Germany, the HBSC study, in combination with studies such as KiGGS, provides information on the health trends of young people nationwide as a fundamental contribution to federal health reporting (GBE) in childhood and adolescence [15]. HBSC results have, for example, been condensed into Fact Sheets for a number of health indicators and uploaded to the GBE website. Overall, the data helps to identify fields of action and risk factors in a targeted manner and provides a basis for decision makers in politics and health practice to initiate health promotion and prevention measures. ‘But data alone will not create change, especially if it does not get into the hands of decision makers who determine funding levels and government priorities’ [38]. HBSC hopes to build a bridge between science and politics and, in close co-operation with decision makers, strengthen the health of children and adolescents [38]. In future, it would therefore be desirable, in close co-operation with the ministries of health and culture, to more strongly establish the HBSC study in the school environment, create incentives to increase participation and develop corresponding structures to continuously implement the study.

Overall, the HBSC results provide important starting points to identify risk groups and fields of action for politics and initiate targeted and needs-oriented measures of prevention and health promotion as well as to corroborate and evaluate the success of these measures through the surveys repeated every four years. HBSC results can provide a basis to determine and monitor health targets and constructively influence the debate on the health development of children and adolescents. Against the backdrop of Germany’s Prevention Act (PrävG), which was adopted in 2015, this is of particular importance.

## Key statements

- The HBSC study is one of the largest global studies on child and adolescent health, and one of the most important data foundations for WHO health reporting within the WHO Europe region.
- HBSC aims to collect and provide data every four years on the health and health behaviour of students aged 11, 13 and 15.
- HBSC is conducted according to standardised, internationally co-ordinated surveying and implementation guidelines across all participating countries.
- HBSC helps identify relevant risk factors and fields of action, and provides a basis to determine focuses for prevention and health promotion, especially in the school setting.
- The results also serve as a basis to determine and monitor health targets, in particular against the backdrop of Germany’s Prevention Act (PrävG).

## Figures and Tables

**Table 1:** Topics within the HBSC questionnaire Source: Inchley et al. (2018) [6], HBSC International Coordinating Centre (2020) [17]

**Compulsory fields of the core questionnaire (mandatory packages)**
▶ **Sociodemographic data** on factors such as sex, age, family structure, migration background, indicators of family social standing (family affluence) ▶ **General health indicators** for example on subjective health and well-being, physical and mental health ▶ **Health and risk behaviour** on factors such as substance use, physical activity, media use, dietary habits, bullying and violence, as well as sexual behaviour (only 15-year-olds) ▶ **Social contexts** for example questions regarding family (i.e. family support), school (class environment, school burdens, support by teachers and students), peers (for example quality of relationships)
**Optional question modules for Germany (optional packages)**
▶ Gender role orientation ▶ Measures to control weight and body satisfaction ▶ Health literacy ▶ Symptoms of depression and stress

**Table 2:** Distribution of the 2017/18 sample by federal state, sex and number of schools* Source: 2017/18 German HBSC study

Federal State	Schools, total (n)	Students, total (n)	Students, total (%)	Students, sex (n)	
				Girls	Boys
Baden-Württemberg	17	562	12.9	330	232
Bavaria	28	989	22.8	505	484
Berlin	5	104	2.4	52	52
Brandenburg	9	181	4.2	92	89
Bremen	2	22	0.5	14	8
Hamburg	3	144	3.3	77	67
Hesse	10	240	5.5	126	114
Mecklenburg-Western Pomerania	3	67	1.5	37	30
Lower Saxony	12	359	8.3	188	171
North Rhine-Westphalia	26	814	18.7	412	402
Rhineland-Palatinate	10	176	4.0	93	83
Saarland	2	22	0.5	7	15
Saxony	6	181	4.2	99	82
Saxony-Anhalt	5	223	5.1	132	91
Schleswig-Holstein	4	141	3.2	68	73
Thuringia	4	122	2.8	74	48
**Total**	**146**	**4,347**	**100**	**2,306**	**2,041**

*Absolute number of cases unweighted, percentages weighted

**Table 3:** Distribution of the 2017/18 sample by sex, type of school and grade (absolute frequencies) Source: 2017/18 German HBSC study

Type of school	5^th^ school year		7^th^ school year		9^th^ school year		Total	
	Girls	Boys	Girls	Boys	Girls	Boys	Girls	Boys
Grammar schools	337	300	335	290	358	289	1,030	879
Intermediate secondary schools	126	108	140	131	155	124	421	363
Mixed forms*	226	212	185	202	271	190	682	604
Lower secondary school	50	73	61	73	62	49	173	195
**Total**	**739**	**693**	**721**	**696**	**846**	**652**	**2,306**	**2,041**

* The mixed form is a heterogeneous group and includes comprehensive schools, schools that combine lower and intermediate secondary education, as well as primary schools in Berlin and Brandenburg.

**Table 4:** Frequencies and percentage distribution (weighted) in the 2017/18 HBSC cycle by sex, age, type of school, migration background and family affluence (n=2,306 girls, n=2,041 boys) Source: 2017/18 German HBSC study

	Girls		Boys		Total	
	N	%	N	%	N	%
**Age group** 11 years 13 years 15 years	720 717 869	31.5 31.7 36.8	676 695 670	33.4 34.7 31.9	1,396 1,412 1,539	32.5 33.2 34.4
**Type of school** Grammar school Other types of school	1,030 1,276	39.5 60.5	879 1,162	37.3 62.7	1,909 2,438	38.4 61.6
**Migration background** None One-sided Two-sided	1,467 266 569	60.6 12.3 27.0	1,300 214 523	61.3 10.8 27.9	2,767 480 1,092	61.0 11.6 27.5
**Family affluence (FAS)** High Medium Low	364 1,337 436	17.0 62.6 20.4	318 1,462 340	15.0 69.0 16.0	682 2,799 776	16.0 65.8 18.2

FAS = Family Affluence Scale
